# Weight gain trajectories patterns from pregnancy to early postpartum: identifying women at risk and timing to prevent weight regain

**DOI:** 10.1186/s12884-022-05154-4

**Published:** 2022-11-04

**Authors:** Cinthya Muñoz-Manrique, Belem Trejo-Valdivia, Sonia Hernández-Cordero, Alejandra Cantoral, Andrea L. Deierlein, Elena Colicino, Megan M. Niedzwiecki, Robert O. Wright, Andrea A. Baccarelli, Martha María Téllez-Rojo

**Affiliations:** 1 Department of Nutrition and Bioprogramming, National Institute of Perinatology, Mexico City, Mexico; 2 Centro de Investigación en Nutrición y Salud, Instituto Nacional de Salud Pública, 3226, Avenida Universidad 655, Santa María Ahuacatitlán, Morelos 62100 Cuernavaca, México; 3 Research Center for Equitable Development EQUIDE, Universidad Iberoamericana, Ciudad de México, México; 4 Health Department, Universidad Iberoamericana, Mexico City, Mexico; 5 Department of Epidemiology, School of Global Public Health, New York University, New York, NY USA; 6 Department of Environmental Medicine and Public Health, Icahn School of Medicine at Mount Sinai, New York, NY USA; 7 Department of Environmental Health Science, Mailman School of Public Health, Columbia University Medical Center, New York, NY USA

**Keywords:** Weight gain, Maternal, Pregnancy, Postpartum, Trajectories, Women of reproductive age

## Abstract

**Background:**

Woman's weight changes during pregnancy and postpartum contribute to obesity and health outcomes later in life. This study aimed to identify and characterize weight change trajectories from pregnancy to one year postpartum among adult women.

**Methods:**

We used data from an ongoing cohort of healthy adult women (*n* = 819) with singleton pregnancies from 2007 – 2011. Sociodemographic data, pre-pregnancy body weight, and sedentary and breastfeeding practices were collected using questionaries applied by trained professionals. We applied a group-based trajectory modeling to distinguish weight change measured in the second and third trimesters of pregnancy and at one month, six, and 12 months postpartum. Multinomial regression models were run to characterize each trajectory.

**Results:**

We identified six weight change trajectories with the main difference in the patterns followed after one month of delivery. One in three women (36.7%) was classified in some of the three postpartum weight gain trajectories and regained weight from the second trimester of the first year postpartum. Women who followed some of these trajectories were more likely to have higher age, obesity before pregnancy, < 10 years of schooling, and partner, compared with women (10.7%, *n* = 87) in a postpartum sustained-fast-lost-weight trajectory (*p* < 0.05).

**Conclusions:**

Women with obesity before pregnancy have higher odds of regaining gestational weight after delivery without reaching their pre-pregnancy weight. The first six months postpartum are crucial to establishing obesity prevention strategies. Further research is needed to evaluate the effect of the interventions that prevent substantial weight gain through reproductive years in high-risk women.

## Background

Overweight and obesity rates have increased worldwide among all age groups [1, 2]. In Mexico, 73% of adult women have overweight or obesity, and the prevalence of obesity has risen significantly in the last few years [3]. Obesity during reproductive age is a metabolic risk factor for adverse perinatal outcomes and non-communicable disease (NCD) later in life [4–6]

The childbearing years can be a time of sustained weight increase in women. Women are expected to gain weight during pregnancy to promote adequate fetal growth and healthy birth weight in newborns [7]. After delivery, women may lose the weight they gained to reach pre-pregnancy weight. However, we can recognize different weight patterns during these moments. Although most women gain weight during pregnancy, the rate of gain differs between them. Some women exceed gestational weight gain recommendations since early pregnancy, while others have insufficient gestational weight [8]. During postpartum, three different patterns appear: some women lose gestational weight gained until they reach pre-pregnancy weight; others may cease weight loss and retain gestational weight gained, and others may go through, along with not reaching pre-pregnancy weight, weight regain during the postpartum period [9–11].

Body weight before, during, and after pregnancy results from a complex interaction between biological, behavioral, and social factors. Maternal factors related to a higher increase in weight during pregnancy and postpartum are pre-pregnancy body mass index, education, socioeconomic status, age, parity, breastfeeding, food intake, physical activity, sedentarism, sleep hygiene, and psychological stress [12–14].

Distinct weight trajectories during reproductive age highlight the variability among women and manifest the need to identify those women at higher risk of obesity or obesity-grade increase. Most research on childbearing weight trajectories has come from the United States and Western Europe [9–11, 15]. In Mexico and Latin America, most of the studies have analyzed weight during pregnancy and postpartum at two-time points instead of identifying weight trajectories [16–18]. Using weight history at multiple time points may be more helpful in capturing the onset and duration of obesity, segregating individuals into distinct life-course weight trajectories, and predicting diseases and mortality rather than a static measures such as gestational weight gain and postpartum weight retention [19].

Pregnancy and postpartum are critical to establishing strategies to impact women’s and children’s health. Studying weight gain throughout these times with a trajectory approach may have an advantage in highlighting timely periods to develop obesity prevention strategies and identifying vulnerable or high-risk groups [20]. This study aimed to identify trajectories of weight change from pregnancy to one year postpartum. We also sought to characterize these trajectories according to sociodemographic data, pre-pregnancy BMI, and postpartum behaviors.

## Methods

### Study sample

This present study is a secondary analysis of data from the ongoing Programming Research in Obesity, Growth, Environmental, and Social Stress (PROGRESS) pregnancy cohort in Mexico City. Details of PROGRESS are published elsewhere [21]. Briefly, pregnant women with less than 20 weeks of gestation were recruited from four prenatal care clinics within the Mexican Social Security Institute (IMSS, by its acronym in Spanish). Women were eligible for participation if they were 18 years or older and planned to reside in Mexico City for the next three years. Exclusion criteria were a cardiovascular or renal disease, use of steroids or antiepileptic drugs, and daily alcohol consumption. Women provided written informed consent. The Institutional Review Boards from the National Institute of Public Health in Mexico, the Icahn School of Medicine at Mount Sinai, and the Harvard TH Chan School of Public Health in the United States (US) approved the study protocol.

Women attended study visits at baseline (< 20 weeks of gestation) and during the third trimester (27–36 weeks of gestation) as well as three times during the first year postpartum (one month, six months, and 12 months postpartum). There were 956 women with live singleton births. For this analysis, we excluded women who had a subsequent pregnancy within the following year of the current pregnancy (*n* = 9), who did not return for follow-up visits (*n* = 8), or who delivered preterm (less than 37 weeks of gestation, *n* = 120). We excluded women with preterm delivery since these women had incomplete weight trajectories and a higher probability of having adverse perinatal outcomes, which may influence weight change patterns [8]. The final analytic sample was 819 women.

### Measures

At baseline, trained social workers administered a questionnaire to collect maternal sociodemographic characteristics. We used the Mexican Association of Marketing Research and Public Opinion Index (AMAI), a 13 × 6 battery based on family possessions, living conditions, and education, to categorize six socioeconomic status (SES) levels ranging from lowest (1) to highest (6) SES within the cohort [22]. Based on the exploratory analysis of its pattern association with weight trajectories, we reclassified these categories as low (1, 2), medium (3), and high (4, 5, 6). Women self-reported their pre-pregnancy weight (kg) six months before pregnancy. We adjusted these self-reported weights using an imputation procedure to account for potential recall bias [23]. Maternal pre-pregnancy weight was predicted using a longitudinal model (linear mixed-effects) with repeated weight data from six months before pregnancy to the third-trimester visit. We used tenfold cross-validation to test the model’s performance based on those women with weights (*n* = 87) close to their last menstrual period (LMP). The root mean squared error of 3.21 kg was considered a measure of predictive accuracy [24]. We calculated pre-pregnancy BMI using adjusted pre-pregnancy weight (kg) and measured height (m) at the baseline visit. Pre-pregnancy BMI was classified using World Health Organization criteria (WHO, 2005): underweight (< 18.5 kg/m^2^), healthy weight (18.5–24.9 kg/m^2^), overweight (25.0–29.9 kg/m^2^), and obesity (≥ 30 kg/m^2^).

Trained personnel measured women’s weight (without shoes and clothes) during pregnancy (baseline and third-trimester visits) and postpartum (one-, six-, and 12-month visits). Weight was measured using a combined mechanical scale and stadiometer (Health-O-Meter; Scaleomatics INC, Cleveland, OH) to the nearest 100 g. Following the Institute of Medicine’s (IOM, 2009) recommendations for gestational weight gain rate [7], we classified women’s gestational weight gain at the second and third trimesters of pregnancy (considering pre-pregnancy BMI and gestational age) as insufficient, adequate, and excessive. Gestational age was defined according to the LMP and corroborated with the Capurro method. We adjusted gestational age weeks in 33 cases where gestational age at birth differed by more than two weeks with LMP.

At each study visit (except 12 months postpartum), women reported the average daily time spent in physical activities, such as walking, and sedentary behaviors, such as watching television or reading. Physical activity was categorized as inactive (< 20 min per day), minimally active (20 – 60 min per day), and active (> 60 min per day). Sedentary behavior was categorized as non-sedentary (< 120 min per day), moderate sedentary (120–180 min per day), and sedentary (> 180 min per day) [25]. At one and six months postpartum, women reported breastfeeding their infant (yes or no) and if they were exclusively providing breast milk (yes or no). Breastfeeding was categorized as not breastfeeding, not exclusive breastfeeding, and exclusive breastfeeding. The intensity of breastfeeding was defined as the daily number of times a woman breastfed her baby at one and six months postpartum.

### Statistical analysis

We first explored the maternal weight’s probability distribution at five points in time: two during pregnancy and three during the first year postpartum. Weeks lapsed were counted since LMP: baseline visit at 18 ± 1.3 weeks (second trimester of pregnancy), 31.7 ± 1.4 weeks (third trimester of pregnancy), 43.7 ± 1.3 weeks (one month postpartum), 65.7 ± 1.3 weeks (six months postpartum), and 92.0 ± 1.5 weeks (12 months postpartum). We transformed weeks into the log scale to deal with the uneven time lapses between consecutive measurements. As part of the exploratory analysis, we also studied the behavior of maternal variables and evaluated for potential selection bias by comparing characteristics between women included and excluded from the analysis.

We constructed the possible weight trajectories and assessed their shape. Group-Based Trajectory Models were used to identify women following similar weight change trajectories [26]. This type of models forms a family of semiparametric statistical techniques used to analyze longitudinal data in clinical fields. This approach helps us distinguish patterns and their appropriate polynomial order beyond a linear association.

We considered a model with time points varying among women, a normal distribution for the response, and trajectory shapes that followed a cubic polynomial. Two to six types of trajectories were evaluated. The optimum number of types of trajectories was chosen based on the lower Bayesian Information Criterion (BIC), probabilities of membership (> 90%), and odds of correct classification (> 5). Given the above optimal number, each woman was classified in the trajectory of her highest probability; thus, women in the sample formed as many groups as types of trajectories identified.

Additionally, we identified the main characteristics of the women’s membership in the respective trajectories using multinomial regression models. We used the first trajectory as a reference since it followed a continuous postpartum weight loss. Because a higher pre-gestational BMI in women could result from older age or higher parity, we tested first and second-order interactions.

## Results

Women had a mean age of 27 ± 5.4 years and 11.8 ± 2.8 years of schooling at enrollment. Most women had at least one child before the current pregnancy (62.7%), reported living with a partner (81.2%), and were classified as low SES (51.8%). The mean pre-pregnancy BMI was 26.7 ± 4.3 kg/m^2^. Prevalence of underweight, healthy weight, overweight, and obesity were 1.2%, 42.7%, 38.5%, and 17.6%, respectively. Out of the women included, 86.6% (*n* = 701), 74.7% (*n* = 612), 67.8% (*n* = 555), and 52.9% (*n* = 434) returned to the visit at third trimester of pregnancy, one month postpartum, six months postpartum, and 12 months postpartum, respectively. Compared with women with weight data for all visits (*n* = 306), women with missing data were similar for most sociodemographic characteristics, except pre-pregnancy BMI. However, there were no differences in the proportions of women with pre-pregnancy overweight or obesity.

**Fig. 1:**
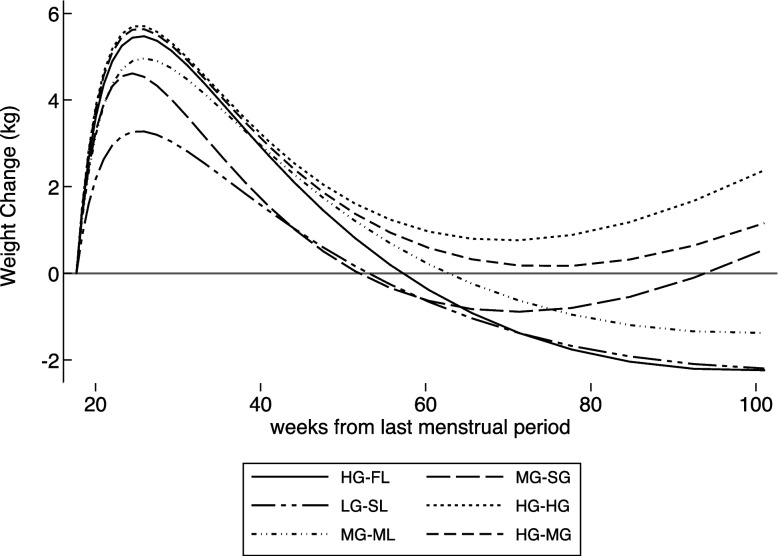
Trajectories of weight change (kg) from the second trimester of pregnancy to one year postpartum. The trajectories were out of phase-based to the average weight of the second trimester of pregnancy, starting at 0 kg (indicating without change). The key denotes the type of trajectories named HG-FL (solid), MG-ML (short dash-dot-dot), LG-SL (long dash-short dash-short dash), HG-MG (dash), HG-HG (very short dash), and MG-SG (long dash). HG-FL, high weight gain during pregnancy and fast weight loss postpartum; HG-HG, high weight gain during pregnancy and high gain postpartum; HG-MG, high weight gain during pregnancy and moderate gain postpartum; LG-SL, lower gain during pregnancy and moderate loss postpartum; MG-SG, moderate weight gain during pregnancy and slow gain postpartum; MG-ML, moderate weight gain during pregnancy and moderate loss postpartum

We identified six distinct trajectories of overall weight change from the second trimester of pregnancy to 12 months postpartum, each with two-time intervals (Fig. 1). The first time interval corresponded with weight change during pregnancy and the first month postpartum. The second interval corresponded with weight change after the first month postpartum. As expected, all trajectories showed weight gain during pregnancy and substantial weight loss in the first month postpartum; however, they differed in the magnitude of such weight change. In the second time interval, two distinct groups could be observed in addition to the differences in the magnitude of weight change. In the first group, women continued to lose weight, while women gained weight in the other group.

We named the trajectories according to the magnitude of weight increase and weight gain (loss or gain) in these two-time intervals observed. Trajectory 1 corresponds to a high weight gain during pregnancy and a fast weight loss along postpartum (HG-FL); trajectory 2 refers to a moderate weight gain during pregnancy and a moderate loss along postpartum (MG-ML); trajectory 3 relates to a lower weight gain during pregnancy and a slow loss along postpartum (LG-SL). In the second group, trajectory 4 corresponds to a high weight gain during pregnancy and a moderate gain postpartum (HG-MG); trajectory 5 corresponds to a high weight gain during pregnancy and a high gain postpartum (HG-HG); and finally, trajectory 6 corresponds to a moderate weight gain during pregnancy and a slow gain postpartum (MG-SG). Most women were classified in trajectory 3 (28.8%), followed by trajectories 2 (23.8%), 4 (20.6%), 5 (11.7%), 1 (10.7%), and 6 (4.4%).

The sociodemographic characteristics of women for each type of trajectory are presented in Table 1, and the RRs of their association based on a multinomial regression model are shown in Table 2. Being in trajectory 1 decreased as pre-pregnancy BMI (kg/m^2^) increased. Compared to women in this trajectory, women in all other trajectories were older (*p* < 0.01). Women with < 10 years of schooling had a lower probability of membership in trajectories 3 or 5 (*p* < 0.01). Also, the probability of membership in trajectory 5 was the lowest if women lived with a partner (*p* < 0.05). We observed a higher probability of membership in trajectory 3 in women with medium and high SES (*p* < 0.10). Women with more than two children previous to their current pregnancy and not working outside the home were more likely to be in trajectory 4 and 6, respectively (*P* < 0.10). We did not observe an interaction between pre-pregnancy BMI, age, and parity on the membership in one trajectory (*p* < 0.30).

**Table 1:** Characteristics of women according to the type of trajectory

	**All women** ***n* = 819**	**1 (HG-FL)** ***n* = 87**	**2 (MG-ML)** ***n* = 195**	**3 (LG-SL)** ***n* = 236**	**4 (HG-MG)** ***n* = 169**	**5 (HG-HG)** ***n* = 96**	**6 (MG-SG)** ***n* = 36**
**Age, years**^a^	27.1 ± 5.5	25.0 ± 4.6	27.1 ± 5.7	27.3 ± 5.4	27.6 ± 5.1	27.5 ± 6.0	27.3 ± 5.1
**Schooling, years**^b^	12 (5)	11 (3)	12 (4)	12 (4)	12 (5)	12 (4)	12 (5.5)
< 10 years	345 (42.1%)	46 (52.9%)	90 (46.1%)	86 (36.4%)	75 (44.4%)	31 (32.3%)	17 (47.2%)
≥ 10 years	474 (57.9%)	41 (47.1%)	105 (53.9%)	150 (63.6%)	94 (55.6%)	65 (67.7%)	19 (52.8%)
**Parity**	2 (2)	2 (2)	2 (2)	2 (2)	2 (2)	2 (2)	2 (2)
Nulliparous	305 (37.2%)	34 (39.1%)	69 (35.4%)	94 (39.8%)	58 (34.3%)	36 (37.5%)	14 (38.9%)
1–2 births	390 (47.6%)	43 (49.4%)	98 (50.3%)	111 (47.0%)	73 (43.2%)	49 (51.0%)	16 (44.4%)
≥ 3 births	124 (15.2%)	10 (11.5%)	28 (14.3%)	31 (13.1%)	38 (22.5%)	11 (11.5%)	6 (16.8%)
**Living with partner**							
Yes	665 (81.2%)	64 (73.6%)	160 (82.1%)	189 (80.1%)	142 (84.0%)	83 (86.5%)	27 (75%)
No	154 (18.8%)	23 (26.4%)	35 (17.9%)	47 (19.9%)	27 (16.0%)	13 (13.5%)	9 (25%)
**Working outside home**							
Yes	557 (68.0%)	59 (67.8%)	122 (62.6%)	175 (74.2%)	120 (71.0%)	63 (65.6%)	18 (50.0%)
No	262 (32.0%)	28 (32.2%)	73 (37.4%)	31 (25.8%)	49 (29.0%)	33 (34.4%)	18 (50.0%)
**Socioeconomic status**							
Low	424 (51.8%)	52 (59.8%)	111 (56.9%)	104 (44.1%)	83 (49.1%)	56 (58.3%)	18 (50.0%)
Medium	307 (37.5%)	27 (31.0%)	68 (34.9%)	97 (41.1%)	72 (42.6%)	28 (29.2%)	15 (41.7%)
High	88 (10.7%)	8 (9.2%)	16 (8.2%)	35 (14.8%)	14 (8.2%)	12 (12.5%)	3 (8.3%)
**Anthropometric**							
Height, m^a^	1.55 ± 0.1	1.50 ± 0.1	1.53 ± 0.1	1.55 ± 0.1	1.56 ± 0.1	1.58 ± 0.1	1.58 ± 0.1
Pre-pregnancy weight, kg^a^	63.2 ± 11.1	47.5 ± 3.8	54.7 ± 3.1	61.5 ± 3.9	69.2 ± 3.7	78.1 ± 4.5	89.3 ± 5.4
Pre-pregnancy BMI, kg/m^2 a^	26.2 ± 4.2	20.1 ± 1.7	23.3 ± 1.8	25.6 ± 2.1	28.4 ± 2.1	31.2 ± 2.6	35.6 ± 3.3
Underweight	10 (1.2%)	10 (11.5%)	0 (0%)	0 (0%)	0 (0%)	0 (0%)	0 (0%)
Healthy weight	359 (42.7%)	77 (88.5%)	164 (84.1%)	98 (41.5%)	9 (5.3%)	2 (2.1%)	0 (0%)
Overweight	315 (38.5%)	0 (0%)	35 (15.9%)	131 (55.5%)	123 (72.8%)	29 (30.2%)	1 (2.8%)
Obesity	144 (17.6%)	0 (0%)	0 (0%)	7 (3%)	37 (21.9%)	65 (67.7%)	35 (97.2%)

Women’s weight was classified using WHO criteria: underweight (< 18.5 kg/m^2^), healthy weight (18.5, 24.9 kg/m^2^), overweight (25.0, 29.9 kg/m^2^), and obesity (≥ 30 kg/m^2^)

*HG-FL* High weight gain during pregnancy and fast weight loss postpartum, *HG-HG* High weight gain during pregnancy and high gain postpartum, *HG-MG* High weight gain during pregnancy and moderate gain postpartum, *LG-SL* Lower gain during pregnancy and moderate loss postpartum, *MG-SG* Moderate weight gain during pregnancy and slow gain postpartum, *MG-ML* Moderate weight gain during pregnancy and moderate loss postpartum

^a^Mean ± SD

^b^Median (interquartile range)

**Table 2:** Relative risks of membership in each trajectory according to sociodemographic characteristics

	**2 MG-ML**^**1**^	**3 LG-SL**^**1**^	**3 HG-MG**^**1**^	**4 HG-HG**^**1**^	**5 MG-SG**^**1**^
**Age, years**	1.08 [1.03–1.14]^2^	1.08 [1.04–1.14] ^2^	1.10 [1.05–1.16]^2^	1.09 [1.04–1.16]^2^	1.09 [1.01–1.18]^2^
**Schooling, years**	1.03 [0.94–1.13]	1.08 [0.98–1.18]^3^	1.08 [0.98–1.17]	1.09 [0.99–1.22]^3^	1.09 [0.95–1.25]
< 10 vs ≥ 10	0.76 [0.46–1.27]	0.51 [0.31–0.84]^2^	0.71 [0.42–1.19]	0.43 [0.23–0.77]^2^	0.79 [0.36–1.73]
**Parity**	1.09 [0.87–1.35]	0.99 [0.80–1.22]	1.17 [0.94–1.47]	1.02 [0.80–1.31]	1.04 [0.74–1.45]
1–2 vs nulliparous	1.12 [0.65–1.93]	0.93 [0.55–1.58]	0.99 [0.56–1.75]	1.07 [0.57–2.01]	0.90 [0.38–2.11]
≥ 3 vs nulliparous	1.37 [0.60–3.16]	1.12 [0.49–2.52]	2.22 [0.98–5.03]^3^	1.03 [0.39–2.75]	1.45 [0.44–4.78]
**Without partner vs partner**	0.61 [0.33–1.10]^3^	0.69 [0.38–1.22]	0.52 [0.28–0.99]^4^	0.43 [0.20–0.92]^4^	0.92 [0.38–2.26]
**Working outside home (yes vs no)**	0.79 [0.46–1.35]	1.36 [0.79–2.32]	1.16 [0.66–2.03]	0.90 [0.48–1.67]	0.47 [0.21–1.05]^3^
**Medium SES vs low SES**	1.17 [0.67–2.05]	1.79 [1.04–3.08]^4^	1.67 [0.95–2.93]^3^	0.96 [0.50–1.84]	1.60 [0.70–3.67]
**High SES vs low SES**	0.93 [0.37–2.32]	2.19 [0.94–5.05]^3^	1.09 [0.43–2.79]	1.39 [0.53–3.67]	1.08 [0.25–4.53]
**Pre-pregnancy BMI, kg/m**^**2**^	2.2 [1.8–2.70]^2^	4.2 [3.3–5.34]^2^	7.6 [5.8–9.9]^2^	12.51 [9.3–16.9]^2^	22.1 [15.4–31.6]^2^

*HG-FL* High weight gain during pregnancy and fast weight loss postpartum, *HG-HG* High weight gain during pregnancy and high gain postpartum, *HG-MG* High weight gain during pregnancy and moderate gain postpartum, *LG-SL* Lower gain during pregnancy and moderate loss postpartum, *MG-SG* Moderate weight gain during pregnancy and slow gain postpartum, *MG-ML* Moderate weight gain during pregnancy and moderate loss postpartum, *SES* Socioeconomic status

^1^ Data are RR [95% CI] from crude multinomial regression models. Group of reference: trajectory 1- High Weight Gain-Fast Weight Lost (HG-FL)

^2^*P* ≤ 0.01

^3^*P* ≤ 0.10

^4^*P* ≤ 0.05

Weight and lifestyle behaviors during pregnancy and postpartum of women for each trajectory are summarized in Table 3. The proportion of women with excessive gestational weight at the second and third trimesters of pregnancy was 1.6% and 26.5%, respectively. These values differed by trajectory, showing that women in trajectories characterized by obesity before pregnancy had the highest proportion of excessive gestational weight gain in at least one of the two moments. On the other hand, the proportion of women with insufficient gestational weight gain was the highest in trajectory 1. Women in trajectory 3 had the highest proportion of adequate gestational weight gain in both moments of pregnancy.

**Table 3:** Weight and lifestyle behaviors during pregnancy and postpartum of women for each trajectory

**Trajectory**	**Time**	**Weight (kg)**	**Adequate GWG**^a^	**Excessive GWG**^a^	**Sedentary behavior**^b^	**No BF**	**BF**	**Intensity of BF (Times/d)**
**1 (HG-FL)**	*2 T*	48.8 ± 3.6	4 (4.6%)	1 (1.2%)	58 (66.7%)			
	*3 T*	54.7 ± 3.1	28 (40.0%)	4 (5.7%)	38 (54.3%)			
	*1 mo*	49.6 ± 3.3			53 (75.7%)	4 (6.5%)	58 (93.6%)	8 (3)^c^
	*6 mo*	47.4 ± 3.5			37 (67.3%)	17 (33.3%)	34 (66.7%)	6 (5)^c^
	*12 mo*	46.7 ± 3.7						
**2 (MG-ML)**	*2 T*	56.2 ± 2.9	19 (9.7%)	2 (1.0%)	140 (71.8%)			
	*3 T*	62.0 ± 3.1	80 (48.8%)	16 (9.8%)	101 (60.5%)			
	*1 mo*	57.0 ± 2.7			109 (74.7%)	9 (6.4%)	131 (93.6%)	8 (4)^c^
	*6 mo*	55.2 ± 3.4			82 (73.2%)	35 (30.7%)	79 (69.3%)	8 (3)^c^
	*12 mo*	54.6 ± 3.3						
**3 (LG-SL)**	*2 T*	62.8 ± 3.6	54 (22.9%)	0 (0%)	158 (66.9%)			
	*3 T*	68.9 ± 3.2	112 (54.9%)	46 (22.6%)	126 (60.9%)			
	*1 mo*	63.6 ± 3.2			137 (75.3%)	14 (8.1%)	158 (91.9%)	8 (4)^c^
	*6 mo*	63.0 ± 3.1			112 (75.2%)	50 (38.5%)	80 (61.5%)	6 (4)^c^
	*12 mo*	62.5 ± 3.6						
**4 (HG-MG)**	*2 T*	70.7 ± 3.3	66 (39.1%)	5 (3.0%)	129 (76.3%)			
	*3 T*	76.8 ± 3.4	62 (44.3%)	57 (40.7%)	80 (56.7%)			
	*1 mo*	71.4 ± 3.1			99 (77.9%)	6 (5.0%)	113 (94.7%)	8 (4)^c^
	*6 mo*	71.0 ± 3.4			95 (84.8%)	29 (28.7%)	72 (71.3%)	7 (4)^c^
	*12 mo*	70.8 ± 4.2						
**5 (HG-HG)**	*2 T*	79.2 ± 4.4	42 (43.8%)	4 (4.2%)	61 (63.5%)			
	*3 T*	85.4 ± 3.6	28 (34.2%)	42 (51.2%)	51 (60.7%)			
	*1 mo*	79.9 ± 3.8			45 (70.3%)	8 (13.6%)	51 (86.4%)	8 (4)^c^
	*6 mo*	80.7 ± 4.5			46 (79.3%)	15 (27.8%)	39 (72.2%)	6 (4)^c^
	*12 mo*	80.1 ± 4.5						
**6 (MG-SG)**	*2 T*	90.9 ± 5.6	18 (50.0%)	1 (2.8%)	28 (77.8%)			
	*3 T*	96.7 ± 4.7	10 (31.3%)	18 (56.5%)	21 (65.6%)			
	*1 mo*	91.5 ± 4.9			18 (78.3%)	2 (11.1%)	16 (88.9%)	8 (3)^c^
	*6 mo*	91.3 ± 5.9			15 (68.2%)	6 (31.6%)	13 (68.4%)	6 (5)^c^
	*12 mo*	91.4 ± 7.3						

*GWG* Gestational weight gain, *No-BF* Not breastfeeding, *BF* Breastfeeding, *HG-FL* High weight gain during pregnancy and fast weight loss postpartum, *HG-HG* High weight gain during pregnancy and high gain postpartum, *HG-MG* High weight gain during pregnancy and moderate gain postpartum, *LG-SL* Lower gain during pregnancy and moderate loss postpartum, *MG-SG* Moderate weight gain during pregnancy and slow gain postpartum, *MG-ML* Moderate weight gain during pregnancy and moderate loss postpartum, *2 T* Second trimester, *3 T* Third trimester, *1 mo* One month postpartum, *6 mo* Six months postpartum, *12 mo* 12 months postpartum

^a^Following IOM’s (2009) Recommendations of Gestational Weight Gain (GWG)

^b^Defined as ≥ 120 min per day watching TV or reading books

^c^Median (interquartile range)

One-third of women breastfed exclusively at one month postpartum, and 1.92% continued at six months postpartum. At both study visits, most women (~ 60%) reported no-exclusive breastfeeding. The median intensity of breastfeeding was eight times per day (interquartile range (IQR) = 3) in the first month and seven times per day (IQR = 5) at six months. At one month postpartum, we observed that women in trajectories 5 and 6 had a higher proportion of not breastfeeding. However, this finding was not observed at six months postpartum, where a higher frequency of not breastfeeding was observed in women belonging to trajectory 3. Regarding sedentary behaviors in postpartum, trajectory 4 showed a higher proportion of sedentary women at one and six months postpartum.

## Discussion

We identified six weight change trajectories running from the second trimester of pregnancy up to one year after delivery in Mexican adult women. All groups increased weight during pregnancy, followed by a substantial loss at one month postpartum; however, the weight change and timing varied according to the six trajectories. The inflection point in the trajectories was the change after one month postpartum, where some women continued to lose weight while others initiated a regain of gestational weight. Trajectories of weight regain were characterized mainly by pre-pregnancy BMI and maternal age. Furthermore, these trajectories were also related to having more than two children before the current pregnancy, lower SES, less than ten years of schooling, having a partner, and not working outside the home.

Clinical recommendations suggest that all pregnant women reach their pre-pregnancy weight and a healthy weight in the coming months after childbirth [27, 28]. In our study, ~ 60% of the women followed some pre-pregnancy weight return pattern in the first postpartum year. In contrast, about one in three women showed a weight regain pattern from three months postpartum. Women with the latter weight trajectories are in a higher-risk group since they may experience significant changes in adipose tissue shortly and later metabolic consequences [29].

Although the factors that impact weight gain after becoming a mother are well documented, especially maternal obesity [30], distinguishing the moment in which weight gain onsets and the characteristics of women at risk give us information for patient-centered care for obesity. In the present study, we identified that women with the same pre-pregnancy BMI followed distinctive weight trajectories and that this was related to certain sociodemographic factors. Being older, having more than two children, and having a partner were characteristics of women who followed postpartum weight regain trajectories. However, the contribution of these factors to weight gain still needs to be clarified, as other studies are not consistent with our findings, especially for parity and social support [31, 32].

Biological, physiological, and psychological causes that happened before, during, and after pregnancy may explain the increased postpartum weight and not returning to pre-pregnancy weight. Our study showed a higher proportion of early excessive gestational weight gain in trajectories with postpartum weight regain. This finding supports the evidence related to excessive gestational weight gain as a predictor of postpartum weight retention [30]. In the same line that pre-pregnancy obesity increases the risk of adverse perinatal outcomes, maladaptive pregnancy changes in these women, especially adipose tissue expandability dysfunction, may have a detrimental effect on postpartum weight [33].

In addition to the potential dendritic impact of inadequate adipose tissue expansion, a return to unhealthy behaviors postpartum may also influence weight regain. Sedentary behavior during postpartum life has been associated with postpartum weight retention, independent of pre-pregnancy BMI [34, 35]. Even though no postpartum weight gain trajectories were characterized by sedentary behaviors such as spending more hours in front of the screen, we observed a higher percentage of women with these behaviors postpartum in the weight gain trajectories.

Breastfeeding practices are another factor related to weight in the postpartum [34–36]. Our findings showed that women in the trajectories with weight regain and slow weight loss after six months postpartum had a higher proportion of cessation of breastfeeding at one and six months postpartum, respectively. These breastfeeding practices may be explained by poor technique and sociodemographic factors such as pre-pregnancy obesity, return to work, and lack of support to maintain breastfeeding [37–39].

Postpartum weight gain may be a contributing factor to the burden of obesity in the Mexican population. In our study, most women with pre-pregnancy obesity (97%) followed some regained weight trajectory. The latter is consistent with the evidence that maternal obesity before pregnancy is a risk factor for body mass increase later in life [30]. According to the latest national health and nutrition survey, by 2020, the prevalence of obesity among adult Mexican women was 40.2%, 2.7 pp more than eight years before [3]. This rapid growth in obesity prevalence manifests a lack of meeting the WHO target of no prevalence increase of adult obesity between 2010 and 2025 [40]. Additionally, beyond the failure to reach pre-pregnancy weight, the weight gain soon after childbirth supports the need to implement obesity prevention strategies with a life course perspective and during sensible times in women.

Beyond the weight change after giving birth, it is essential to evaluate the consequences of this change on health. In another type of analysis from this cohort, Soria et al. found that women with postpartum weight retention and weight gain at 12 months had a higher BMI, weight circumference, and insulin resistance at six years postpartum than women who returned to their pre-pregnancy weight [9]. The relation between fat mass accumulation during pregnancy and postpartum and cardiovascular risk factors in later years was also reported in women from Project Viva and Danish National Birth Cohort [10, 11]. Postpartum maternal weight change trajectories are also associated with children with higher weight for height and energy intake among the Mexican population [41].

Our study has some strengths and limitations. One of the strengths of this study is that we measured and analyzed weight prospectively from pregnancy to one year postpartum at different points. These prospective repeated measures allowed us to study the timing of weight change, contrary to the current literature on the topic, which cannot identify the timing of weight change [30]. However, one limitation of our study was that the research team could not measure pre-pregnancy weight, influencing the trajectories starting in the second trimester of pregnancy. To overcome this limitation, we used predicted weight to address the bias associated with weight underreporting and misclassification. Although we included women without postpartum data to run a group-based trajectory model, we may have needed to include other factors that characterize each trajectory. However, we only observed differences in pre-pregnancy BMI between women with complete and incomplete data. Our findings do not apply to women with a lower duration of gestation (< 37 weeks) or adverse perinatal outcomes.

## Conclusions

Interventions to prevent women’s obesity during pregnancy and postpartum should be addressed as a continuous process. Efforts should be directed to establish timely and effective pre-conception and inter-pregnancy interventions in women with overweight or obesity to influence the cycle of obesity and chronic diseases To support and promote healthy lifestyle behaviors, interventions and strategies also need to consider women’s characteristics influencing weight retention and weight gain. Other studies are required to validate the reproducibility of these trajectories and explore how these trajectories are related to the risk of diseases in later stages.

## Data Availability

The datasets used and/or analyzed during the current study are available from the corresponding author on reasonable request.

## Abbreviations

HG-FL: High weight gain during pregnancy and fast weight loss postpartum
HG-HG: High weight gain during pregnancy and high gain postpartum
HG-MG: High weight gain during pregnancy and moderate gain postpartum
LG-SL: Lower gain during pregnancy and moderate loss postpartum
MG-SG: Moderate weight gain during pregnancy and slow gain postpartum
MG-ML: Moderate weight gain during pregnancy and moderate loss postpartum
